# An investigation of psychological responses to COVID-19 in Irish healthcare workers: longitudinal quantitative and nested qualitative study

**DOI:** 10.12688/hrbopenres.13204.1

**Published:** 2021-02-03

**Authors:** Donal G. Fortune, Helen L. Richards, Andrew Wormald, Kieran O Connor, Margaret McKiernan, Pablo Najt, Amanda O Dwyer, Edmond O Dea, Paul Burke, Joseph Eustace

**Affiliations:** 1 Psychology, University of Limerick, Limerick, Ireland; 2 Clinical Psychology, HSE Mid West Community Healthcare, Limerick, Ireland; 3 Clinical Psychology, Mercy University Hospital, Cork, Ireland; 4 Medicine, Mercy University Hospital, Cork, Ireland; 5 Nursing, Mercy University Hospital, Cork, Ireland; 6 Clinical Education and Research Centre, University of Limerick Hospitals Group, Limerick, Ireland; 7 Medicine and HRB CRF, University College Cork, Cork, Ireland

**Keywords:** COVID-19, Psychological Phenomena and Processes, Health Occupations, Environment and Public Health

## Abstract

COVID-19 is an unprecedent occurrence in modern times and individuals who work within healthcare settings, face a broad array of challenges in responding to this worldwide event. Key information on the psychosocial responses of such healthcare workers (HCWs) in the context of COVID-19 is limited and in particular there is a need for studies that utilise longitudinal methods, an overarching theoretical model, and use of a cohort of participants within a defined geographical area across acute and community settings. The work packages making up the current research project use quantitative and qualitative methods to examine the psychological sequelae for HCWs in the context of COVID-19 in geographically adjacent healthcare areas (South and Mid-West of Ireland) across four time points (induction, 3 months, 6 months, and 1 year follow-up). The quantitative arm of the project (WP 1) utilises the Common-Sense Model of Self-Regulation (CSM-SR) and examines a number of key psychological factors pertinent to this model including perceptions about COVID-19 and infection more generally, coping, formal and informal support and a number of impact variables including mood, sleep quality, and perceptions of stigma. The qualitative study (WP 2) will address HCWs experiences of working during the pandemic, ascertain any additional areas of psychological functioning, environmental and workplace factors and resources that may be utilised by HCWs and that are not assessed by the quantitative study protocol, focusing particularly on those staff groups typically underrepresented in previous studies.

## Introduction

Following the outbreak of SARS-CoV in 2002 and MERS-Cov in 2012, SARS CoV-2 (COVID-19) is the third coronavirus to have resulted in significant outbreaks within the past 20 years, and is certainly the most significant in terms of its worldwide reach, rates of infection and death. The absence of vaccine roll-out has in general led to more behaviourally focused national policies based upon establishing and promoting behaviours such as hand washing, social distancing, and limiting social contacts to reduce transmission and protect the ability of the health service to provide effective clinical care. 

Obviously individuals working in healthcare settings (healthcare workers, HCWs), are central to the management of the healthcare impact of COVID-19, and because of the nature of that work, HCWs have an increased likelihood of exposure. In a large register-based study comprising the entire Scottish healthcare workforce, patient facing HCWs were three times more likely to be admitted to hospital with COVID-19 than non-patient facing HCWs ( Shah *et al*., 2020). In addition to the perception of heightened risk, individuals working in healthcare settings including healthcare professionals, administrative and support staff have had to adapt to the stresses of responding to often abrupt and significant changes to everyday work practices ( Kennelly *et al*., 2020; Shapiro, 2020).

HCWs share many of the fears and concerns held by individuals in the general population ( Krishnamoorthy *et al*., 2020; Sheraton *et al*., 2020). In this context, it is unsurprising that systematic reviews of research data have demonstrated high levels of distress in HCWs with one in five HCWs experiencing significat anxiety and depression and approximately two in five reporting insomnia ( Pappa *et al*., 2020); similar results were reported by Serrano-Ripoll *et al*. (2020), and Muller *et al*. (2020), who also urge caution given the risk of bias, heterogeneity and imprecision in the reported data from their review. 

There is some evidence that distress in HCWs during COVID-19 may be higher than in the general public ( Chew *et al*., 2020; Shechter *et al*., 2020), which perhaps may be understood within the context of moral distress ( Borges *et al*., 2020), fear of infection and perceptions of stigma ( Ramaci *et al*., 2020; Taylor *et al*., 2020). In this context, and while there have been helpful phased approaches developed to support psychological well-being of HCWs (e.g., Billings *et al*., 2020; Tomlin *et al*., 2020; Williams *et al*., 2020), it is likely that there will be a number of short and longer term mental health challenges for HCWs. A systematic review and meta-analysis on HCWs across a number of recent virus outbreaks including COVID-19 suggested that compared to controls, staff who were in contact with affected patients had higher levels of psychological distress in addition to acute and post-traumatic stress symptoms ( Kisely *et al*., 2020). Risk factors for psychological distress in this study, included being younger, being at an earlier stage of their career, being parents of dependent children, having reduced practical support, and holding higher perceptions of stigma. In terms of COVID-19 specifically, HCWs who were described as frontline healthcare workers had higher trauma or distress scores than non-frontline healthcare workers ( Alshekaili *et al*., 2020; Cai *et al*., 2020; Kang *et al*., 2020; Lai *et al*., 2020; Lu *et al*., 2020; Maiorano *et al*., 2020; Rossi *et al*., 2020). In some studies there were differences in how distress was being expressed: for example in the Alshekaili *et al*. (2020) study, there were no significant differences in depression scores between patient facing and non-patient facing HCWs, however HCWs in the frontline group were 1.5 times more likely to report experiencing levels of stress, insomnia and anxiety. By contrast there are also studies which found that the differences in distress between frontline and non-frontline HCWs were very small ( Babore *et al*., 2020), or were not significantly different ( Jahrami *et al*., 2020; Man *et al*., 2020), or that reported that frontline HCWs had significantly lower distress (vicarious traumatisation) than non-frontline HCWs ( Li *et al*., 2020). Indeed, predicting outcomes from broad categories of frontline HCWs or patient facing HCWs is complex and is probably subject to a number of environmental factors. For example, working with patients diagnosed with COVID-19 in intensive care units is associated with higher distress in HCWs ( Azoulay *et al*., 2020; Sharma *et al*., 2020); however, such working environments tend not to be associated with an increased risk of HCW infection ( Eyre *et al*., 2020) possibly due to the nature of infection protocols within this setting and viral load of patients at that later infection/admission stage. It is also likely that other psychological factors may have prominence in relation to understanding distress, such as communication, support and stigma ( Sharma *et al*., 2020).

Some research suggests that the general public significantly overestimate the probability that HCWs are carriers of SARS-CoV2. In a recent study in the US and Canada, almost one third of participants held the belief that HCWs were likely to have COVID-19; 39% believed that HCWs who treat COVID patients should be isolated; while 35% believed such HCWs should be separated from their families ( Taylor *et al*., 2020). Thus while there were visible demonstrations of support for HCWs (e.g. public applause etc.), stigmatising attitudes towards HCWs also exist and probably co-exist with the kinds of observed public demonstrations of support for HCWs. HCWs perceptions of stigma may be an important component of their psychological response to working during COVID-19.

While much can be learned from previous virus outbreaks, and from cross sectional or brief follow-up studies, we do not yet know how HCWs respond to COVID-19 challenges over time; nor do we know the interplay between HCWs personal beliefs or perceptions about COVID-19, coping, stigma, the nature of their healthcare working environment, and the use of formal and informal resources that may buffer the potential psychological effects on well-being. While the nature of the pandemic and its mental health effects have encouraged mental health professionals to be very active and nimble about providing supports, we do not yet know what may prove helpful in terms of intervention. A recent Cochrane review suggested that there is a lack of evidence from studies carried out during pandemics that may prove helpful in informing the choice of mental health interventions for HCWs ( Pollock *et al*., 2020). Similarly there is recent evidence of a potential mismatch between the organisational sources of HCW distress during COVID-19 and the individualised kind of responses offered by such healthcare systems ( Muller *et al*., 2020).

In addition to the scarcity of current data-driven interventional approaches, few studies have utilised a coherent theoretical model in which to understand the experiences of HCWs in the context of COVID-19. The Common-Sense Model of Self-Regulation (CSM-SR) is a well-established health behaviour model ( Leventhal *et al*., 1980) that has relevance to the experience of HCWs in the context of COVID-19. The CSM-SR is a model of how individuals identify possible illness threat, and initiate and maintain their self-regulation in the face of such threat. The CSM-SR proposes a number of key representations of illness that may be held by individuals including Illness identity (label and symptoms), potential consequences, timeline (duration and course of illness), beliefs about the extent to which the condition can be cured or controlled by the person or by treatment, whether the symptoms associated with the condition makes sense to the individual (coherence), and personal beliefs about the causes of the illness. The key suggestion of the CSM-SR model is that aspects of the individual’s perception of illness guide their selection of coping behaviours which may be directed towards the cognitive and/or emotional representations of the illness, and which in turn may predict psychological and behavioural outcomes ( Hagger *et al*., 2017; Leventhal *et al*., 1984). 

There are very few studies that have examined changes in illness perceptions over time, or how such changes may influence psychosocial outcomes, and to our knowledge there are currently no published longitudinal studies using the CSM-SR in HCWs in the context of COVID-19. A cross-sectional study by Man *et al*. (2020) on 67 HCWs, found that while distress and stress were high among their sample, total illness perceptions score (sum of all perception items) and coping scores did not differ between HCWs who were working in COVID-19 receiving settings versus those who were not. Shahzad *et al*. (2020), examined illness perceptions within an integrated stressor-strain outcome and agonistic behaviour model (defence, avoidance and aggression) in a cross-sectional study on 345 paramedical staff, utilising summed scores of illness perceptions to represent perceived emotional and perceived cognitive threat from COVID-19. They found significant relationships between stronger perceptions of cognitive and emotional threat from COVID-19 and depression, physiological anxiety and emotional exhaustion, and that this led to agonistic behaviour. Moreover, perceived social support was a moderator of anxiety, depression, and emotional exhaustion on agonistic behaviour.

Given the research needs outlined above, the principal aim of this project is to utilise the CSM-SR to investigate the role of illness perceptions and coping and support resources on psychological impact factors, and to investigate how such factors may change over time. The project will gather longitudinal quantitative and qualitative data across four time points, and provide key data on the nature of psychological, social and environmental factors for healthcare staff wellbeing in the context of COVID-19, that may prove helpful for supporting HCWs.

## Methods

Ethical approval was granted by the Clinical Research Ethics Committee of the Cork Teaching Hospitals and HSE Limerick Hospitals ethics committee (CREC: ECM 4 (a) 09/04/2020 & ECM 3 (u) 09/04/2020 and UHL REC 041/2020). Local site approval was obtained for each of the individual hospitals and community health area participating in the study.

### Aims

Our aim is to examine a number of key psychological factors and resources inmpacting health care staff in the context of COVID-19 within two work packages across four time points (induction, 3 months, 6 months and 1 year).

### Objectives

1) To recruit a sample of participants (HCWs) working in a healthcare context in two adjacent regions; the South and Midwest regions of Ireland. 

2) To enable participants to remotely complete a questionnaire assessment of mood, coping, post-traumatic stress symptoms, beliefs about infection and COVID-19, stigma, sleep, and the availability and use of support resources. 

3) To undertake interviews with 50 participants.

4) To follow up participants from both WP 1 and WP 2 at 3 months, 6 months and 1 year in relation to completion of quantitative measures and interviews.

### Inclusion criteria

Inclusion and exclusion criteria are common across WP 1 and WP 2.

HCW is defined for purpose of this project as an individual working within the structure of the health service. These roles will include staff working in COVID-19 receiving hospitals, including staff that have either direct contact with COVID-19 patients or no contact with such patients, staff working in non-COVID-19 receiving hospitals, and staff working in the community, whether directly with patients who have been diagnosed with COVID-19 or not. In addition to healthcare professionals, we include administrative, clerical, portering, domestic and catering staff within our definition of individuals working within the health service.

### Exclusion criteria

Absence of consent to study procedures and insufficient fluency in the English language to complete questionnaire assessments or participate in interviews.

### Participants

Participants will be recruited from University of Limerick Hospital Group, HSE Mid-West Community Healthcare, and Cork Teaching Hospitals Group (Mercy University Hospital, South Infirmary Victoria Hospital, Cork University Hospital, Kerry University Hospital, Bantry General Hospital, Mallow General Hospital).

### Procedure

HCWs will be contacted by posting the study description/information sheet on the hospital/community intranet and via an individual email sent to all HCWs. We will also advertise the study on staff notice boards so as to reach staff who may not automatically have access to email/intranet, for example, porters, catering, healthcare assistants etc. For those who wish to participate, a link will be provided to the consent form and study measures which will be hosted online via the Qualtrics platform. The program will not permit participants to proceed in the study if they have not clicked on the consent agreement. There is a separate additional consent for participation in WP2 such that participants may choose to take part in either or both work packages. The online method will also be used for consent to participate in WP2 (qualitative interviews).

The data will not be irrevocably anonymized at source given the need to follow up participants. Data will be coded through a procedure where participants will generate their own code (Self-Generated Identification code - SGIC) which will permit their follow-up data points to be paired using their unique SGIC. We will use a four-question SGIC. Participants will be requested to report their mother’s first initial of their first name, their number of older brothers, the month in which they were born, and the first letter of their own middle name. Thus, a participant who indicates that their mother’s name was Mary (M), that they have one older brother (01), were born in July (07), and whose middle name is Louise (L) would self-generate their identification code as M0107L. This will be encrypted and data locked between assessment points. The unique SGIC will permit automatic matching with previous assessment data points, and is considered an effective participant follow-up method ( Audette *et al*., 2020).

### WP 1. Quantitative study

Our principal research questions are based on the self-regulation model of human behaviour ( Leventhal *et al*., 1980). Specifically we are interested in:

RQ1) What is the relationship between representations of illness, coping and support and psychological impact; 

RQ2) What are the rates of distress in the sample and change in distress levels over the course of COVID-19;

RQ3) How do illness beliefs/perceptions, coping, support and impact variables change over time in response to formal (e.g., practical organisational support, use of psychological first aid, use of employee assistance programme, peer support, HSE online stress control etc.) and informal supports.

RQ4) Are such changes in line with the self-regulation model.

***Study measures.*** We are using brief and ultra-brief assessments of the key variables of interest to this study to reduce the load on participants.

*Event impact - Mood*

The Patient Health Questionnaire-4 (PHQ-4) ( Kroenke *et al*., 2009) is an ultra-brief measure of current symptoms of anxiety and depression. The four-item measure consists of a two-item anxiety scale (GAD-2) (score range, 0-6), combined with a two-item depression scale (PHQ-2) (score range, 0-6). The PHQ-4 is a well validated measure.

*Event impact - Trauma*

The Primary Care PTSD Screen (PC-PTSD-5 ; Prins *et al*., 2016) will be used as an indicator of the impact of the event in terms of symptoms of trauma over the past month. The PC-PTSD is a 4-item screener, with one question reflecting each symptom cluster. The PC-PTSD-5 has demonstrated excellent validity ( Prins *et al*., 2016).

*Event impact - Sleep quality*

The Pittsburgh Sleep Quality Index (PSQI; Buysse *et al*., 1989) is a 19 item measure of sleep quality and regular sleep habits. The measure has been well validated and has diagnostic specificity of 86.5% and sensitivity of 89.6% in categorising good and poor quality sleepers ( Buysse *et al*., 1989).

*Event impact - Stigma*

The Stigma Scale for Chronic Illness (SSCI-8) was developed as a short form of the SSCI ( Molina *et al*., 2013). The SSCI-8 consists of 5 items designed to evaluate enacted stigma and 3 to evaluate internalized stigma. Each item is rated on a five-point Likert scale that ranges from “1” (never) to “5” (always) and total scores range from 8 to 40. Higher scores indicate higher levels of stigma. The internal consistency (Cronbach’s alpha) of the SSCI- 8 is good (0.89).

*Illness perceptions about COVID-19*

The Brief Illness Perceptions Questionnaire ( Broadbent *et al*., 2006) is a 9 item self-reported assessment of people’s beliefs across a range of illness experiences. One item each assesses the dimensions of Consequences, Timeline, Personal Control, Treatment control, Illness Identity, Concern, Understanding/Coherence, and Emotional response. Causal beliefs are assessed via a free text format whereby an individual is asked to provide up to three causes. Higher scores represent a stronger belief in particular illness perceptions.

*Perceived vulnerability to disease contagion*

The perceived vulnerability to disease contagion measure ( Duncan *et al*., 2009) is a 15 item scale which assesses people’s self-reported susceptibility to disease and germ avoidance. For example, “In general, I am very susceptible to colds, flu, and other infectious diseases. Responses are rated on a 7-point scale from 0 (strongly disagree) to 6 (strongly agree), higher scores reflecting higher perceived susceptibility.

*Coping*

The Brief Resilient Coping Scale (BRCS; Sinclair & Wallston, 2004) is a four item scale which focuses on adaptive ways of coping with stress. It has been shown to have good reliability and validity. Scores range from 1 “does not describe me at all” to 5 “describes me very well”. Normative data are available. 

*Social support*

Social support will be measured with the 12-item Multidimensional Scale of Perceived Social Support ( Zimet *et al*., 1988). The scale is a self‐report measure and distinguishes PSS from three sources: family (4 items; e.g. “My family is willing to help me make decisions”), friends (4 items; “My friends really try to help me”) and a significant other (4 items; “There is a special person who is around when I am in need”). Respondents answer through a 7‐point scale for each item. Internal reliability of the MSPSS in healthcare workers is good with α = >0.90 ( Hamama *et al*., 2019).

***Data management.*** All personally identifiable data will be subject to the General Data Protection Regulation (GDPR) and 2018 Data Protection Act. Participation in the research project and the identity of the participants will be treated as confidential, and no patient identifiable records or results relating to the study will be disclosed to any third party other than the authorised investigators. A user self-generated identification code (SGIC) will be developed by each participant (as above), and this number will be mapped to identifiable participant details in the form of an encryption key, held securely away from the data. Data will be stored on an encrypted password protected computer. All identifiers will be removed from the data at the point of data entry.

***Data analysis quantitative data: power and analytic approach.*** For our principal research question, we intend to utilise a bias corrected bootstrap mediation model to examine whether coping mediates the relationship between illness perceptions and the psychological impact of COVID-19 as predicted by the CSM-SR. In terms of sample size estimation, and using Cohen’s criteria (1988) for small, medium and large effect sizes, we expect that the effect of illness perceptions on coping will be of medium size (0.39), the effect of coping on psychological impact will be small (0.14), and coping will completely mediate the effect of illness perceptions on psychological impact. Therefore, the bias corrected bootstrap mediation model ( Fritz & MacKinnon, 2007), would require a minimum of 391 participants for 0.8 power. Given the likelihood of attrition over the waves of the study, we aim to recruit in excess of this number.

Descriptive statistics will be used to describe the characteristics of the sample (e.g. gender, age, work role etc.). Univariate analyses will utilise t-tests and analysis of variance models for continuous data, and Chi², Fishers Exact test as appropriate will be utilised to examine the association between categorical variables cross sectionally e.g. gender, work role, home circumstances) and logistic regression models will examine change in the distress levels over time. Pearson’s correlations and regression models will enable examination of associations, and analysis of variance models will be used to examine simple change in measured variables over time. We will model the principal longitudinal data using mediation models as outlined above using the 95% bias corrected bootstrap intervals with 5000 bootstrap samples. Indirect effects will be considered significant if the confidence interval does not include zero. 

### WP 2: Qualitative study

We aim to conduct a telephone or teleconferencing interview with 50 participants who agree to this component of the research at the same four time points to ascertain any additional areas of psychological functioning and resources that may be utilized by staff and that are not assessed by our protocol. ****

RQ1) What support strategies are available and utilised by HCWs across the timeline of COVID-19?

RQ2) How are the support strategies experienced by staff, and in what ways are they related to unwanted psychological outcomes?

RQ3) What other supports might HCWs find helpful across the timeline of the pandemic?

RQ4) Are there any additional areas of psychological functioning and resources that may be utilised by staff and that are not assessed by our quantitative protocol.

***Data analysis qualitative data.*** Qualitative data will be analysed using reflexive thematic analysis ( Braun & Clarke, 2006; Braun & Clarke, 2020). The analysis will take a realist perspective, reporting the experiences, meaning and reality of participants ( Braun & Clarke, 2006). A deductive approach to analysis will be used, generating any themes related to psychological wellbeing and supportive care needs in the interviews. The analysis will follow the six stages for thematic analysis outlined by Braun & Clarke (2006), familiarisation with the data, generating initial codes, searching for themes, reviewing themes, defining and naming themes and production of the final report. We will utilise established frameworks for quality ( O’ Brien *et al*., 2014).

## Dissemination

The project will make its anonymised data available. Data availability will be concordant with the open data initiative and the principles of FAIR data management. Measures in the data management plan will be outlined to support data sharing.

A range of additional methods will be utilised to ensure effective dissemination commensurate with the statement on data sharing during public health emergencies. For example:

1) Information and data regarding the project will be provided in the form of social media as appropriate and bulletins for staff groups.
2) Data relating to progress and final results will be presented across the various stakeholder HCW group. In addition, opportunities for presentations/webinars e.g. University/hospital, Community, and national organisational level will also be undertaken.
3) Data will be prepared for publication in peer reviewed journals across disciplines. Primary publications will comply with the HRB open publication policy.

## Study status

WP1 and WP 2 ongoing: follow-up January 2021.

## Discussion

Research on HCWs in the context of COVID-19 suggest that as a group they may be at increased risk for psychological distress. Such distress may arise from the transactional nature of psychological processes, social and workplace factors involved in the nature of their work. HCWs negotiation of complex dilemmas involving for example balancing professional roles with protecting themselves and family members from exposure ( Borges *et al*., 2020), may give rise to significant challenges. COVID-19 is a dynamic event that is likely to show change in terms of reactions, impact and help seeking over time. While there are a number of studies of supports ongoing (e.g., Azizoddin *et al*., 2020; Weiner *et al*., 2020), and systematic reviews of approaches that may prove helpful ( Muller *et al*., 2020; Pollock *et al*., 2020), there is a continued need for approaches to be data driven, useful, inclusive of specific workplace issues, and responsive to the needs of HCWs as events change.

### Limitations

Unlike other studies using on-line data capture methods where HCWs status is self-report and which therefore may be somewhat unreliable, this study uses closed staff email addresses which may be considered to increase the accuracy and reliability of the sampling frame. Nonetheless the study uses a convenience sample relying on a self-selected group who choose to participate. Secondly, the study relies on self-report data, which may lead to bias in reporting. Thirdly, the study may not be generalisable beyond the study sample and Irish HCW population.

## Conclusion

Quantitative and qualitative data from this project will help to provide information across 4 time points on the nature of key psychological, social and organisational/environmental factors for HCWs in the context of COVID-19; the utility of the CSM-SR as a frame in which to understand the role of illness threat and coping/support resources on psychological impact factors, and how they may change over time. It may also provide information on what factors in particular, may help to safeguard the well-being of HCWs, and may also contribute to the nature of interventions to help manage the impact on HCWs of such large scale health emergencies in the future.

## Data availability

No data are associated with this article
