# Supplementary material for: An investigation of psychological responses to COVID-19 in Irish healthcare workers: longitudinal quantitative and nested qualitative study
Source: HRB Open Res. 2021 Feb 3;4:15. [Version 1] doi: 10.12688/hrbopenres.13204.1 (PMC8150119; doi:10.12688/hrbopenres.13204.1)
Supplement: Peer review history

# Peer review: Reviewer response for version 1

**DOI:** 10.21956/hrbopenres.14353.r29500

**Published:** 2021-05-25

**Authors:** Esther Murray

**Affiliations:** 1 Centre for Medical Education, Barts and The London School of Medicine & Dentistry, Queen Mary University of London, London, UK

This longitudinal research project looks both interesting and useful. I am pleased to see the theoretical underpinnings of the CSM presented here. I have a couple of queries:

- Why measure stigma for chronic illness? I can't see it's relevance here since COVID-19 is (usually) an acute illness. Is there not a more suitable questionnaire e.g. for infectious disease?
- Do you think that the answers you receive for vulnerability to contagion will be affected by the fact these are HCW? Has the questionnaire been validated in this population?
- The questions for the qualitative interviews don't seem to be included here - I am guessing they cover the areas laid out as RQs?

Is the study design appropriate for the research question?

Yes

Is the rationale for, and objectives of, the study clearly described?

Yes

Are sufficient details of the methods provided to allow replication by others?

Yes

Are the datasets clearly presented in a useable and accessible format?

Not applicable

Reviewer Expertise:

Health Psychology

I confirm that I have read this submission and believe that I have an appropriate level of expertise to confirm that it is of an acceptable scientific standard.

# Peer review: Reviewer response for version 1

**DOI:** 10.21956/hrbopenres.14353.r28830

**Published:** 2021-03-12

**Authors:** Samir Al-Adawi

**Affiliations:** 1 Department of Behavioral Medicine, College of Medicine and Health Sciences, Sultan Qaboos University, Muscat, Oman

Thank you for considering me to review this study protocol entitled “An investigation of psychological responses to COVID-19 in Irish healthcare workers: longitudinal quantitative and nested qualitative study”. The authors aim to employ both qualitative and quantitative theoretical models to quantify ‘challenges over time in terms of psychological response to COVID-19 among’ Health Care Workers (HCWs). As correctly noted by the authors, many studies have highlighted the rising tide of poor mental health outcomes among HCWs. It is not clear whether such magnitude constitutes acute reaction or something that has refractory nature. Indeed, other social negative ramifications of the pandemic yet to be studied. Therefore, the study protocol poses relevant empirical questions since the magnitude of poor mental health outcomes tends to be higher than the general population and it is not clear whether factors such as individual, organizational, or, for that matter situational, contribute to the observed higher rate of mental distress among HCWs. Similarly, scant attention has been forthcoming regarding the durability of the observed tribulation among HCWs. This study protocol appears to be equipped to address both vertical as well as horizontal aspects of the consequence of COVID-19. The entrance of the common-sense model of self-regulation increases the scientific merit of this protocol. Some minor comments are highlighted below for the authors’ consideration.

**Title**

 I think the title provides the reader with what to expect from the rest of the manuscript. However, to make it more succinct here is my suggestion: “Longitudinal investigation of psychological responses to COVID-19 in Irish healthcare workers: a Mixed-Methods Study”.

**Abstract**

 The abstract provides the required snapshot of the content of the manuscript. I would suggest that the authors could round up the abstract by stating the implication of this protocol. What empirical question this study is likely to answer and specifically how the use of mixed methods supplants the existing literature?

**Introduction**

 The authors have reviewed relevant literature to show the gap in the literature on the durability of the effect of COVID-19. Overall, the authors have done very well in laying the groundwork for the motivation of the present study.

 The stated aims of the study (…” CSM-SR to investigate the role of illness perceptions and coping and support resources on psychological impact factors, and to investigate how such factors may change over time”.) minimize the quantitative aspect of the study. Please consider expanding the aims. 

**Method**

 I think I will have the bulk of my comments will be on the Method section. First of all, it is not clear why this statement appears in the method (“… Our aim is to examine a number of key psychological factors and resources impacting health care staff in the context of COVID-19 within two work packages across four-time points (induction, 3 months, 6 months and 1 year). Suggestion: put it back at the end of the introduction”).

 Some of the subheadings in the METHOD need to be re-arranged and their context fine-tuned. For brevity, the authors could utilize the method recommended by STROBE checklists (https://www.strobe-statement.org/index.php?id=available-checklists). Even though this study utilizes mixed methods, STROBE checklists for observational study (quantitative part of the study – WP1) would help the reader to glimpse the breadth and depth of this interesting study. Hence, to the reader replicate it.

 The authors intend to employ various screening measures (e.g. Patient Health Questionnaire-4”, Primary Care PTSD Screen, Pittsburgh Sleep Quality Index, Stigma Scale for Chronic Illness, Brief Illness Perceptions Questionnaire, Perceived Vulnerability to Disease Contagion Measure. etc. As the authors did not highlight the psychometric properties of these among HCWs, they should at least say that their internal validity will be explored.

 I would think the study flowchart will be an added asset to include.

 Any statement on statistical power or sample size calculation?

 A subheading covering ethical consideration is needed. Plus, a statement is needed on what the authors will do if some of the participants do endorse emotional distress. This is a standard practice narrative such statement.

**Limitations**

 This statement needs to be made more succinct (“Unlike other studies using on-line data capture methods where HCWs status is self-report and which therefore may be somewhat unreliable, this study uses closed staff email addresses which may be considered to increase the accuracy and reliability of the sampling frame.”). 

**References**

 Most of the references recent and relevant.

**Minor Issues**

 Could you please define WP1 and WP 2? If accepted acronym, please cite the relevant citation.

 Rather than ‘Event impact – Mood’, since is about anxiety and depression, may I suggest here: “Event impact – Affective ranges”’

 Please consider whether this statement is still valid (“The absence of vaccine roll-out…”). Recently vaccines are spreading in the market.

 These statements might require rephrasing “…be very active and nimble about…”, “… brief and ultra-brief assessments”. I think the expressions are sophisticated from the eyes of non-native speakers. However, sometimes the authors appear to be tilting towards over-reliance on bombastic words. Maybe another colleague could re-read the manuscript and render it more towards scientific writing.

Is the study design appropriate for the research question?

Yes

Is the rationale for, and objectives of, the study clearly described?

Yes

Are sufficient details of the methods provided to allow replication by others?

Partly

Are the datasets clearly presented in a useable and accessible format?

Not applicable

Reviewer Expertise:

Public Health, Mental Health

I confirm that I have read this submission and believe that I have an appropriate level of expertise to confirm that it is of an acceptable scientific standard.
